# Cellular and antibody response in GMZ2-vaccinated Gabonese volunteers in a controlled human malaria infection trial

**DOI:** 10.1186/s12936-022-04169-8

**Published:** 2022-06-17

**Authors:** Odilon Nouatin, Javier Ibáñez, Rolf Fendel, Ulysse A. Ngoa, Freia-Raphaella Lorenz, Jean-Claude Dejon-Agobé, Jean Ronald Edoa, Judith Flügge, Sina Brückner, Meral Esen, Michael Theisen, Stephen L. Hoffman, Kabirou Moutairou, Adrian J. F. Luty, Bertrand Lell, Peter G. Kremsner, Ayola A. Adegnika, Benjamin Mordmüller

**Affiliations:** 1 Centre de Recherches Médicales de Lambaréné, BP : 242, Lambaréné, Gabon; 2 Institute of Tropical Medicine, University Tübingen, Wilhelmstr. 27, 72074 Tübingen, Germany; 3 German Centre for Infection Research (DZIF), Partner Site, Tübingen, Germany; 4 Département de Biochimie Et de Biologie Cellulaire, Faculté des Sciences et Techniques, Université d’Abomey-Calavi, Cotonou, Bénin; 5 Center of Tropical Medicine and Travel Medicine, Department of Infectious Diseases, Amsterdam University Medical Centers, Amsterdam, The Netherlands; 6 Department for Congenital Disorders, Statens Serum Institut, Copenhagen, Denmark; 7 Centre for Medical Parasitology at Department of International Health, Immunology and Microbiology, University of Copenhagen, Copenhagen, Denmark; 8 Department of Infectious Diseases, Copenhagen University Hospital, Rigshospitalet, Denmark; 9 Sanaria Inc., Rockville, MD 20850 USA; 10 Centre d’Etude et de Recherche sur le Paludisme Associé à la Grossesse et a l’Enfance, Calavi, Bénin; 11 MERIT, Université de Paris, Paris, IRD France; 12 Department of Medicine I, Division of Infectious Diseases and Tropical Medicine, Medical University of Vienna, Vienna, Austria; 13 Department of Parasitology, Leiden University Medical Centre (LUMC), 2333 ZA Leiden, The Netherlands; 14 Fondation pour la Recherche Scientifique, 72 BP45 Cotonou, Bénin; 15 Department of Medical Microbiology, Radboud University Medical Center, Nijmegen, The Netherlands; 16 Cluster of Excellence: EXC 2124: Controlling Microbes to Fight Infection, Tübingen, Germany

**Keywords:** GMZ2, Cytokine, Memory B cells, P. falciparum, CHMI, Microarray

## Abstract

**Background:**

Antibody and cellular memory responses following vaccination are important measures of immunogenicity. These immune markers were quantified in the framework of a vaccine trial investigating the malaria vaccine candidate GMZ2.

**Methods:**

Fifty Gabonese adults were vaccinated with two formulations (aluminum Alhydrogel and CAF01) of GMZ2 or a control vaccine (Verorab). Vaccine efficacy was assessed using controlled human malaria infection (CHMI) by direct venous inoculation of 3200 live *Plasmodium falciparum* sporozoites (PfSPZ Challenge). GMZ2-stimulated T and specific B-cell responses were estimated by flow cytometry before and after vaccination. Additionally, the antibody response against 212 *P. falciparum* antigens was estimated before CHMI by protein microarray.

**Results:**

Frequencies of pro- and anti-inflammatory CD4^+^ T cells stimulated with the vaccine antigen GMZ2 as well as B cell profiles did not change after vaccination. IL-10-producing CD4^+^ T cells and CD20^+^ IgG^+^ B cells were increased post-vaccination regardless of the intervention, thus could not be specifically attributed to any malaria vaccine regimen. In contrast, GMZ2-specific antibody response increased after the vaccination, but was not correlated to protection. Antibody responses to several *P. falciparum* blood and liver stage antigens (MSP1, MSP4, MSP8, PfEMP1, STARP) as well as the breadth of the malaria-specific antibody response were significantly higher in protected study participants.

**Conclusions:**

In lifelong malaria exposed adults, the main marker of protection against CHMI is a broad antibody pattern recognizing multiple stages of the plasmodial life cycle. Despite vaccination with GMZ2 using a novel formulation, expansion of the GMZ2-stimulated T cells or the GMZ2-specific B cell response was limited, and the vaccine response could not be identified as a marker of protection against malaria.

***Trial registration*** PACTR; PACTR201503001038304; Registered 17 February 2015; https://pactr.samrc.ac.za/TrialDisplay.aspx?TrialID=1038

**Supplementary Information:**

The online version contains supplementary material available at 10.1186/s12936-022-04169-8.

## Background

Malaria remains one of the leading causes of maternal and infant mortality in the world [1]. The tools currently available for malaria control include vector control, chemoprophylaxis, prompt diagnosis and use of effective anti-malarial drugs [1]. In addition to existing tools, an effective malaria vaccine would be a game changer for elimination and eradication programmes [2]. Many malaria vaccine candidates have been tested, including GMZ2, a recombinant protein vaccine candidate that targets the asexual blood stages of *Plasmodium falciparum*. GMZ2 comprises a combination of Glutamate-Rich Protein (GLURP) and Merozoite Surface Protein 3 (MSP3) expressed in *Lactococcus lactis* [3]. It has been tested in several studies and has proven to be immunogenic in terms of vaccine-specific IgG production and specific memory B cell generation when using aluminum hydroxide as adjuvant [4–6]. A multicentre Phase II randomized, controlled trial in malaria endemic regions showed significant but low efficacy of the GMZ2-Alhydrogel formulation, ranging from 3.6 to 23% [7]. The latter result raised the question of whether the choice of Alhydrogel as adjuvant is optimal. Particularly, inducing pro-inflammatory T cell-mediated responses could be advantageous. The cationic adjuvant formulation (CAF01) is an adjuvant that has already been used in clinical trials to induce CD8^+^ cytotoxic T lymphocytes against HIV-1 (human immunodeficiency virus-1) peptides [8], and to promote long-lived *Mycobacterium tuberculosis*-specific CD4^+^ T-cell responses [9].

Cytokine producing CD4^+^ T cells have been shown to play an important role in protection against *P. falciparum* infection following immunization with the malaria vaccine RTS, S [10–14]. Moreover, polyfunctional T cells have been associated with higher protective efficacy after vaccination [15–18]. Thus, CAF01 was chosen as a novel adjuvant partner for GMZ2. One of the goals of using CAF01 as an adjuvant for the GMZ2 vaccine candidate was also to enhance the memory response. Memory B cells (MBCs) produce antibodies of switched isotypes with higher affinity [19], and their development and maintenance is modulated by *P. falciparum* infection [20, 21].

In the current study, two regimens of GMZ2 (30 µg and 100 µg), adjuvanted with CAF01 and one regimen (100 µg) of GMZ2 adjuvanted with Alhydrogel were used to investigate in a randomized, controlled, double-blind, phase 1 clinical trial the safety, tolerability, immunogenicity and vaccine efficacy [22]. To estimate the protective efficacy, the study population was challenged using viable cryopreserved *P. falciparum* sporozoites in a controlled human malaria infection (CHMI) by direct venous inoculation (DVI). As reported previously, none of the vaccination regimens could improve protection against malaria infection, and the elicited humoral immune response was not predictive for protection. Nevertheless, surprisingly, the level of antibody levels against the antigen GMZ2 before the vaccination could predict protection against CHMI [22].

Here, the frequencies of cytokine-producing CD4^+^ T cells, the circulating GMZ2-specific B cells following immunization, and the antibody response to a range of over 200 *P. falciparum* antigens were evaluated. The potential of these biomarkers to predict protection against infection or clinical symptoms after CHMI was assessed.

## Methods

### Study design and population

Assessment of intracellular cytokine producing CD4^+^ T cells and B cell responses after vaccine antigen GMZ2 stimulation was nested within a Phase 1 trial aiming to assess the safety, immunogenicity, and efficacy of GMZ2 adjuvanted with CAF01 in fifty Gabonese adults with lifelong exposure to malaria. As part of the inclusion criteria, all participants were tested negative for hepatitis B virus (HBV), hepatitis C virus (HCV), human immunodeficiency viruses (HIV) as well as for malaria parasites at baseline. Detailed information concerning the study design is given elsewhere [22]. Standardized CHMI using the parasite strain *P. falciparum* NF54 was conducted by direct venous inoculation of PfSPZ Challenge (Sanaria Inc.), as described previously [23–25]. Vaccine efficacy was defined as protection from clinical symptoms of malaria or protection from a parasitaemia above 1000 parasites per µl. Peripheral blood mononuclear cells (PBMC) were isolated from venous blood collected at inclusion (D0), and again 28 days after the third vaccination (D84) and were used for immunological assays. Plasma isolated from blood collected at C-1 (one day before the CHMI) was used to quantify the antibody titre against several *P. falciparum* antigens, and to measure the antibody breadth.

### Blood collection, PBMC isolation and stimulation

The different steps of PBMC isolation after sample collection, and of PBMC stimulation are described in detail elsewhere [26]. Briefly, after counting, cells were suspended in the culture medium and stimulated for 18 h with either the vaccine antigen (GMZ2, 4 µg/ml, Henogen S.A. Belgium) or staphylococcal enterotoxin B (SEB, 1 µg/ml, Sigma-Aldrich) as positive control. Unstimulated cells as negative control were incubated in culture medium alone. The culture was done in the presence of anti-CD28/CD49d antibodies (BD Biosciences). Two hours after PBMC stimulation, 1 µg/ml of Golgi Plug (BD Biosciences) was added. Each plate used for stimulation included PBMC isolated both on D0 and on D84 from the same participant. The working conditions remained stable for all samples.

### Intracellular cytokine staining (ICS)

Isolated PBMCs were stained first with Aqua live/dead (Life Technologies) and incubated at room temperature in the dark for 30 min. After washing, they were suspended in 50 μl of filtered Flow Cytometry Buffer FCB (1X PBS (Gibco), 0.5% BSA (Sigma-Aldrich) 2 mM EDTA (Thermo Fischer Scientific)) and 50 μl of Staining Buffer (FCB 2% Beriglobin (CSL Behring)) and stained with anti CD3-PercP Cy5.5 (eBioscience), anti CD4-FITC, (BD Biosciences) followed by an incubation at 4 °C in the dark for 30 min. Anti-IL-10-PE, anti-IL-13-BV711, anti-IL-2-BV785, anti-IFNγ-BV421, anti-TNFα-BV605 and anti-IL4-PE CF594 (all Biolegend) intracellular staining was done according to manufacturer’s recommendations (BD Biosciences). Cell acquisition was performed using a Spectral Cell Analyzer cytometer SP6800 (Sony Biotechnology) 15-color cytometer, with 100,000 cells per tube as the total number of cells acquired. Polyfunctional CD4^+^ T cells were defined as CD4^+^ T cells expressing any combination of IFN-γ, IL-2 or TNF. IL-10, IL-4 or IL-13 producing CD4+ T cells are defined as CD3+CD4+IL-10+, CD3+CD4+IL-10−IL-4+ and CD3+CD4+IL-10−IL-13+, respectively.

### GMZ2 labelling and circulating GMZ2-specific memory B cell staining

Molecular Probes’ Alexa Fluor^®^ 647 Protein Labeling Kit was used to label GMZ2 vaccine antigen with the Alexa Fluor 647 dye as described by the manufacturer. Briefly, the GMZ2 solution was added to the vial of reactive dye containing a magnetic stir bar. The solution was mixed to fully dissolve the dye, and the reaction mixture was stirred for 1 h at room temperature. Then, the provided purification resin was stirred thoroughly to ensure a homogeneous suspension, and the resin was pipetted into the column allowing excess buffer to drain away into a beaker. The reaction mixture was then loaded onto the column. The reaction was rinsed with elution buffer. The elution buffer was added slowly to elute the labelled protein. Two colored bands were observed representing the labelled protein and the unincorporated dye respectively. The labelled protein was collected into a provided collection tube. The reliability of the achieved labelling was tested by spectroscopy before storing at 4 °C for further use.

After thawing, PBMCs were rested overnight at 37 °C and 5% CO_2_ in a cell incubator. Then, cells were stained with Aqua live/dead (Life Technologies) at room temperature in the dark for 30 min. Additionally, circulating GMZ2-specific memory B cells were stained using anti-CD20-BV570 (Biolegend), anti-CD27-BV421 (BD Biosciences), anti-IgG-PE (Biolegend), and the recombinant protein GMZ2 coupled to AF647 (AF647 from Life Technologies) for 30 min at 4 °C in the dark, washed twice and resuspended in 300 µl of flow cytometry buffer to be acquired by the Spectral cell analyzer SP6800. In total, 300,000 cells were acquired per sample and GMZ2-specific B cells were further characterized within the CD20^+^IgG^+^CD27^−^ and CD20^+^IgG^+^CD27^+^ populations.

### Cell count estimation and assay optimization

Blood counts were obtained from Centre de Recherches Médicales de Lambaréné (CERMEL). Total lymphocyte counts were used to calculate the estimated cell counts per phenotype according to their frequencies following both T and B gating strategies (Additional file 1: Fig. S1a and S1b). For both T cell and B cell assays, all time points per volunteer were measured in a single experiment after several optimization tests, namely the calibration of the flow cytometer, the titration of the monoclonal antibodies, the titration of the stimulating antigen, determination of the optimal stimulation period and the assay was repeated with a positive control several times to ensure assay stability.

### GMZ2-specific IgG concentration measurement

The anti-GMZ2 total IgG was measured by enzyme-linked immunosorbent assay (ELISA) on isolated plasma collected at D0 and at D84 as described by Esen et al. [5] with minor modifications. These modifications consisted of the dilution of the plasma sample in PBS, 3% non-fat milk, 0.1% Tween 20, and the use of peroxidase conjugated goat anti-human IgG (Invitrogen) at a 1:65,000 dilution [22]. As reference for the assay, European malaria-naive pooled sera was taken as negative control whereas the pooled sera from Gabonese adults was used as positive control.

### Microarray assay

Protein microarray-based assessments of antibody reactivity against *P. falciparum* antigens were performed as described before with some modifications [27]. Microarrays were produced as described previously at the University of California Irvine, Irvine, CA [28]. In total, 251 *P. falciparum* proteins were expressed using an *Escherichia coli* lysate in vitro expression system and spotted on a 16-pad ONCYTE AVID slide, representing 212 *P. falciparum* antigens. The antigens spotted on the array are summarized in the Additional file 2: Table S2. Secondary antibodies (goat anti-human IgG QDot_®_800) were obtained from Grace Bio-Labs, Inc., (Bend, OR).

The plasma samples from the Gabonese donors were taken one day before challenge by phlebotomy and stored at  − 80 °C. In addition, plasma samples from European donors were obtained from malaria-naïve study participants in CHMI trials performed in Tübingen, Germany. For use on the microarray, plasma samples were diluted 1:100 in 0.05X Super G Blocking Buffer (Grace Bio-Labs Inc.) containing 10% *E. coli* lysate (GenScript, Piscataway, NJ) and incubated for 30 min on a shaker at room temperature (RT). Meanwhile, microarray slides were rehydrated using 0.05X Super G Blocking buffer at RT. Subsequently, rehydration buffer was removed, and samples added on the slides. Samples were incubated for 2 h at RT on a shaker (180 rpm). Afterwards, diluted plasma samples were removed, and microarrays washed using 1X TBST buffer (Grace Bio-Labs, Inc.). Subsequently, secondary antibodies (anti-human IgG Q800, Grace Biolabs, #110,635), were applied at a dilution of 1:250 and incubated for 2 h. After a final washing step, slides were dried by centrifugation at 500*g* for 10 min. Slide images were taken using an ArrayCAM^®^ Imaging System (Grace Bio-Labs Inc.) using the ArrayCAM 400-S Microarray Imager Software.

### Statistical analysis

#### Microarray data analysis

Microarray data were exported from the Imaging software and further analysed using the R statistical software package version 3.6.2. All images were manually checked for any noise signal. Image quality was very high, but rare blurry spots were removed from further analysis. Background correction was performed according to the maximum likelihood estimation for the normal-exponential convolution model [29] using the saddle-point approximation (available in the limma package v3.28.14). Subsequently, data was normalized by log2-transformation. Finally, median normalization was performed to normalize the different assays for background activity of antibodies binding to *E. coli* lysate using mock expression spots. Analysis of plasma antibody levels in the groups with different study outcomes were analysed by Student’s t-test, and the respective p value and fold-change differences of antigen-specific antibody level means were given. Antibody breadth was estimated by comparing the antibody levels of the vaccinated subjects to a cohort of malaria naïve volunteers. For each of the subjects, the number of antibody levels with higher than four-fold the reactivity of the mean of the respective reactivity of the malaria-naïve population was enumerated and defined as the individual antibody breadth.

In addition, the vaccine-specific antibody response at baseline was tested for correlation with the individual antigens spotted on the protein microarrays using Student’s t-test. Heatmaps, box plots and volcano plots were generated using the gplots, ggplot and PAA packages, respectively.

#### Flow cytometry data analysis

Flow cytometer data was analysed using FlowJo Version 10, graphs and statistical analysis were done using GraphPad Prism Version 6, and R (R Core Team (2017), R Foundation for Statistical Computing, Vienna, Austria).

Proportion of activated CD4+ T cells were gated as CD3+ CD4+ cytokine+. Data are expressed as substraction of the gated unstimulated from the antigen-specifically stimulated sample, including subsequent normalization using the average of the positive control stimulation. Flow cytometry data showing erratic staining or samples with viability (Live/dead staining) below 70% were excluded from the analysis for both ICS and B-cell staining.

## Results

### Baseline characteristics of the study population

**Fig. 1:**
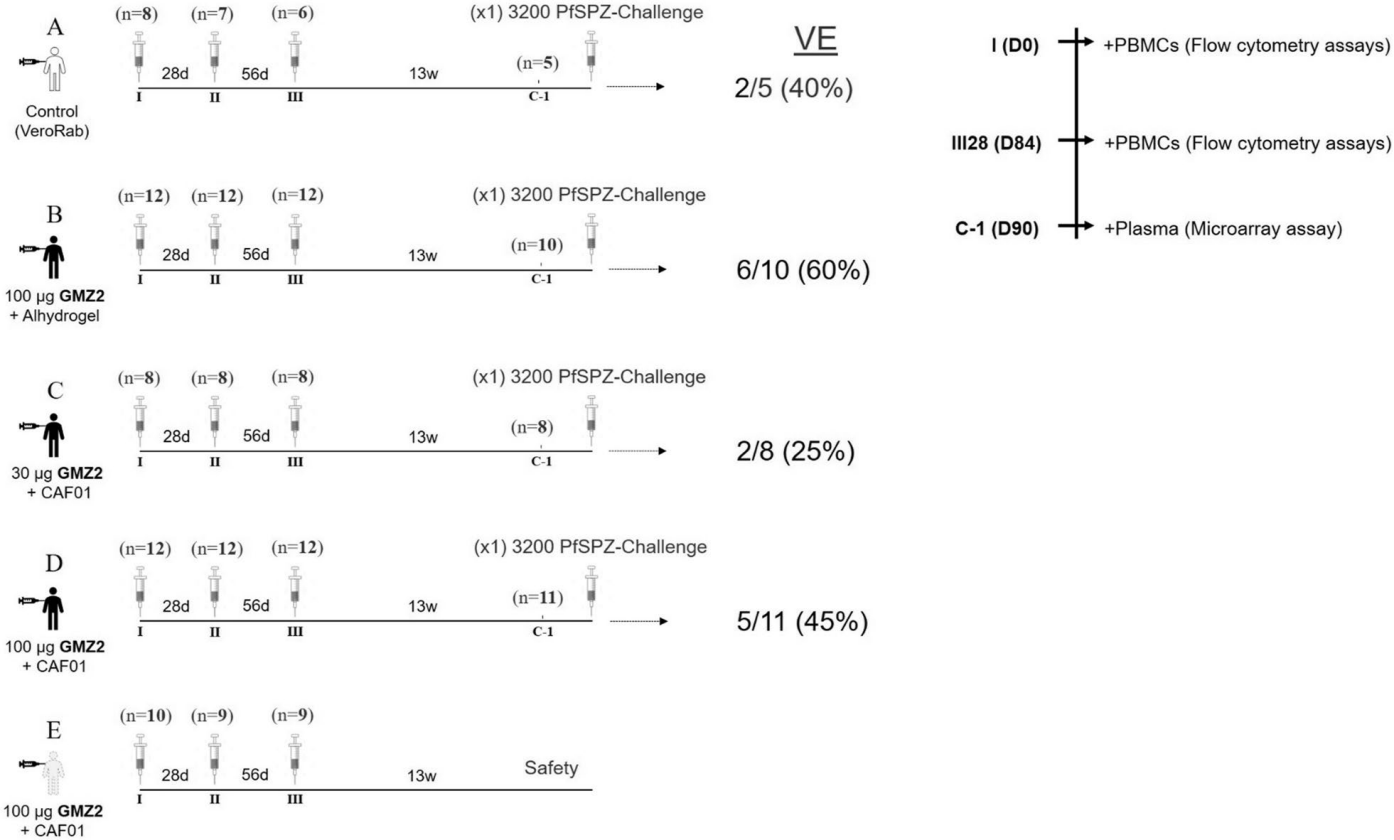
GMZ2-CAF01 study overview. Participants were allocated in five separate groups: (1) Group **A** (control vaccine; n = 8, (2) Group **B** (100 µg GMZ2-Alhydrogel; n = 12), (3) Group **C** (30 µg GMZ2- CAF01; n = 8), (4) Group **D** (100 µg GMZ2-CAF01; n = 12), and (5) Group **E** (100 µg GMZ2-CAF01, without subsequent CHMI; n = 10). All injections were administered intramuscularly in the deltoid muscle on study days 0, 28, and 56, in alternating sides. Thirteen weeks after receiving last completion of the immunization dose, volunteers of Groups A–D underwent CHMI by direct venous inoculation (DVI) of 3200 aseptic, purified, cryopreserved *P. falciparum* sporozoites (Sanaria® PfSPZ Challenge), strain NF54, to assess vaccine efficacy (VE). Group E volunteers were followed-up for 6 months post–immunization without CHMI. Small graph in the upper side corner indicates sampling time points selected to characterize T, B, and antibody breadth responses. Figure legend to the right indicates the time points designed to sample study subjects

The timeline of the study design is depicted in Fig. 1. Baseline characteristics were similar between the groups (Table 1). From the 50 participants included in this study, sufficient PBMC samples for all analyses were available from 44 participants. Blood samples collected before the first immunization (D0), and one month after the last immunization (D84) were used for this study. Among these participants whose PBMC samples were available, six volunteers received a control vaccine (Verorab), 11 received 100 μg GMZ2-Alhydrogel, 7 received 30 μg GMZ2-CAF01 and 20 received 100 μg GMZ2-CAF01. Results of the clinical trial have been published before [22]. The vaccine efficacy (VE) for the treatment arms is summarized in Fig. 1, none of the experimental vaccinations improved protection against the challenge in comparison to the rabies control vaccine significantly.

**Table 1:** Baseline characteristics of the study population

	Rabies	100 µg GMZ2 Alum	30 µg GMZ2 CAF01	100 µg GMZ2 CAF01	p value
Number	N = 6	N = 11	N = 07	N = 20	
Age (years) *	23 [22–33.75]	25 [23–26]	22 [20–25]	22 [19–33]	0.451
Body mass index (kg/m2) ^#^	21.90 (2.74)	23.02 (2.82)	21.78 (1.43)	22.15 (2.04)	0.635
Hemoglobin (g/dl) ^#^	13.73 (1.35)	14.63 (1.31)	13.51 (1.68)	13.84 (1.29)	0.309
White blood cells (cells/µl) *	5.70 [5.3–6.25]	4.90 [4.2–7]	5.30 [4.3–6.7]	5.10 [4.1–6.9]	0.726
Lymphocytes (cells/µl) *	2.17 [1.9–2.3]	2.32 [1.8–2.6]	2.59 [1.8–2.8]	1.74 [1.5–2.5]	0.396
Monocytes (cells/µl) *	0.54 [0.4–0.6]	0.44[0.3–0.6]	0.49 [0.3–0.5]	0.47 [0.3–0.6]	0.507
Neutrophils (cells/µl) *	2.49 [2–2.9]	2.32 [1.2–2.6]	1.82 [1.3–2.5]	1.84 [1.2–2.5]	0.420
Eosinophils (cells/µl) *	0.27 [0.16–0.86]	0.17 [0.11–0.94]	0.61 [0.12–0.91]	0.34 [0.24–0.94]	0.821
Basophils (cells/µl) *	0.03 [0.02–0.08]	0.06 [0.03–0.11]	0.05 [0.02–0.07]	0.05 [0.02–0.06]	

*N*  number of subjects

^*^Median and [Interquartile range], Kruskal–Wallis test

^#^ Mean (Standard Deviation), ANOVA

### GMZ2 stimulated CD4^+^ T cells response

Cytokine-producing CD4^+^ T cells were gated on viability and surface markers as well as intracellular cytokine pattern (Additional file 1: Fig. S1a).

**Fig. 2:**
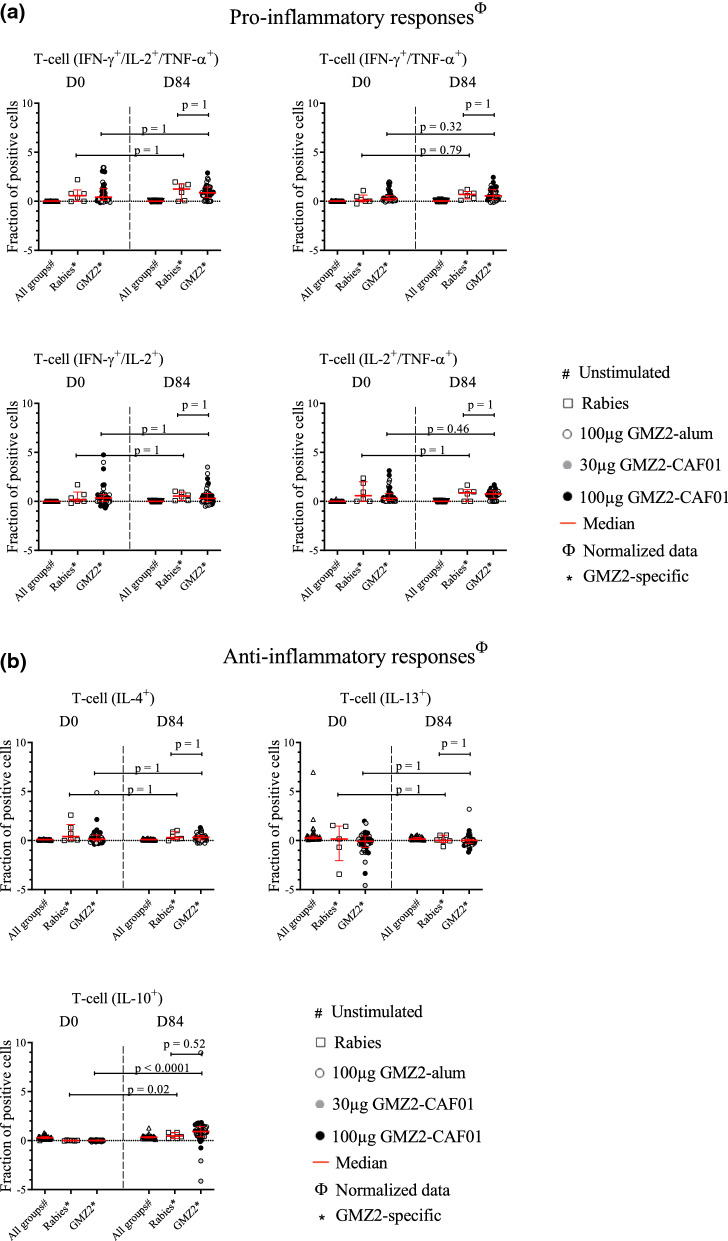
CD4^+^ T cell frequencies following immunization. Isolated PBMCs were stimulated with either medium alone, the vaccine antigen GMZ2, or Staphylococcal enterotoxin B (SEB) as positive control. Thereafter, intracellular cytokine staining was performed, and the cells measured by flow cytometry. Data are expressed after subtraction of unstimulated cell frequencies from that of stimulated with the positive control (SEB), and with GMZ2, and normalization with the average of positive control values. The comparison of the pro-inflammatory cytokine producing CD4^+^ T cells (Fig. 2a), and the anti-inflammatory cytokine producing CD4^+^ T cells (Fig. 2b) between D0 and D84 was performed in those receiving the control vaccine and in those vaccinated with GMZ2 (including those vaccinated with 100 µg GMZ2-Alhydrogel (opened dots), 30 µg GMZ2-CAF01 (grey dots), 100 µg GMZ2-CAF01 (black dots) using Wilcoxon test with Bonferroni correction for multiple comparisons. p value less than 0.05 is considered as statistically significant. All time points per volunteer were measured in a single experiment after several optimization tests, and individual volunteers were measured in separate experiments. Symbols represent individual samples. Red lines represent the median values with interquartile range.

Cytokine (IFN-γ, TNF, IL-2, IL-10, IL-13, and IL-4) production of CD4^+^ T cells was assessed by measuring the fraction of single and multiple cytokine-positive cells following in vitro stimulation with medium alone (mock stimulation), GMZ2 antigen, or with the positive control (SEB). Stimulation with positive control SEB reached stimulation levels of 2–4%, which has been reported similarly before [30, 31]. As expected, most participants circulating pro-inflammatory CD4^+^ T cells reacted to the stimulation with the vaccine antigen GMZ2 at baseline (Fig. 2a), most likely due to their lifelong natural exposure to malaria parasites. All CD4^+^ T pro-inflammatory cytokine combinations had similar values at D0 and D84, regardless of the intervention (i.e., GMZ2 or control vaccine). To increase statistical power during analyses, all groups of those who received the GMZ2 vaccine candidate were pooled. Nevertheless, no difference between the GMZ2 vaccinated groups and the control group on D84 was found.

The GMZ2 antigen stimulated IL-4^+^ and IL-13^+^ CD4^+^ T cell frequencies were similar in the Rabies vaccinated as well as in GMZ2 vaccinated groups before and after vaccination, whereas the proportion of total IL-10 producing CD4^+^ T cells increased significantly in the GMZ2 vaccinated group (45-fold, 95% CI = 20 – 71), as well as in the control group (20-fold, 95% CI 5–35) following vaccination (Fig. 2b). Besides, the proportion of IL-10^+^ CD4^+^ cells were not statistically different between GMZ2- and control-vaccinated groups at D84. Interestingly, additional analyses using estimated blood counts showed an increase in the number of double (IFN-γ^+^/TNF-α^+^ or IL2^+^/TNF- α^+^) and triple positive proinflammatory cytokine (IFN-γ^+^/IL2^+^/TNF-α^+^) producing CD4^+^ T cells after stimulation with GMZ2. In addition, also total number of IL4^+^ and IL10^+^ anti-inflammatory T-cells, but not IL13^+^ T-cells increased in cell number after vaccination (Additional file 1: Fig. S2).

### Circulating B cell response to GMZ2

**Fig. 3:**
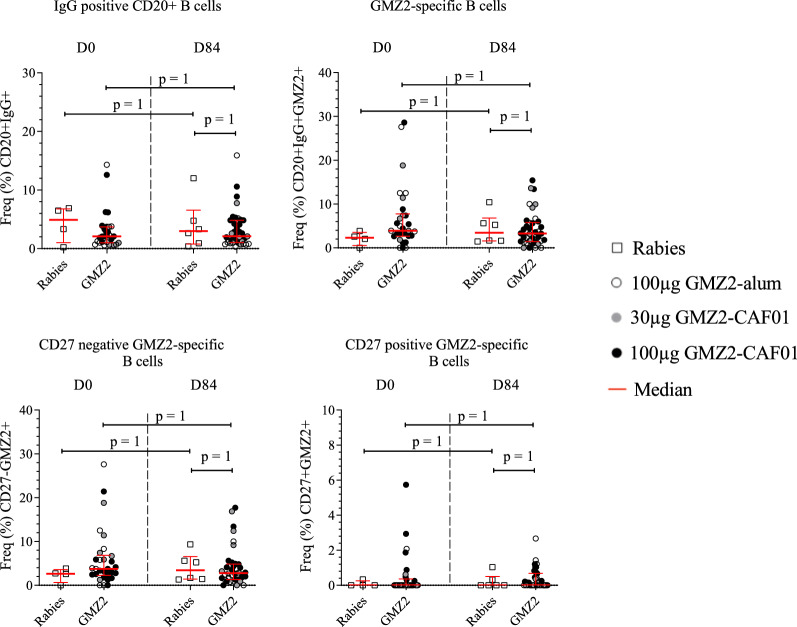
GMZ2-specific B cell frequencies following immunization. B cells were estimated using cryopreserved PBMCs without additional stimulation. The comparison of the GMZ2-specific memory B cells frequencies between D0 and D84 was performed in those receiving the control vaccine and in those vaccinated with GMZ2 (including those vaccinated with 100 µg GMZ2-Alhydrogel (opened dots), 30 µg GMZ2-CAF01 (grey dots), 100 µg GMZ2-CAF01 (black dots) using Wilcoxon test with Bonferroni correction for multiple comparisons. p value less than 0.05 is considered as statistically significant. All time points per volunteer were measured in a single experiment after several optimization tests, and individual volunteers were measured in separate experiments. Symbols represent individual samples. Red lines represent median values with interquartile range

GMZ2-specific B cells were gated on viability and surface markers as well as reactivity to GMZ2 (Additional file 1: Fig. S1b). Here, the generation of circulating GMZ2-specific memory B cells after vaccination was determined by flow-cytometric estimation of antigen-specific CD20^+^ IgG^+^ B cells. Data showed no increment in the CD20^+^IgG^+^ cell frequency in all GMZ2 vaccinated individuals at D84. Similarly, CD20^+^IgG^+^ cell frequency in response to the vaccine antigen GMZ2 remained without changes and neither CD27^+^ nor CD27^−^ GMZ2-specific cell frequencies were associated with any increment post vaccination (Fig. 3). Interestingly, estimated cell counts of the total lymphocyte count numbers showed slightly higher CD20^+^IgG^+^ counts at D84 (p < 0.05). However, GMZ2-specific CD20^+^IgG^+^ cells did not increase after immunization (Additional file 1: Fig. S3).

### T and B cells stimulated post-immunization with the vaccine antigen GMZ2 are not associated with protection against clinical malaria in CHMI

**Fig. 4:**
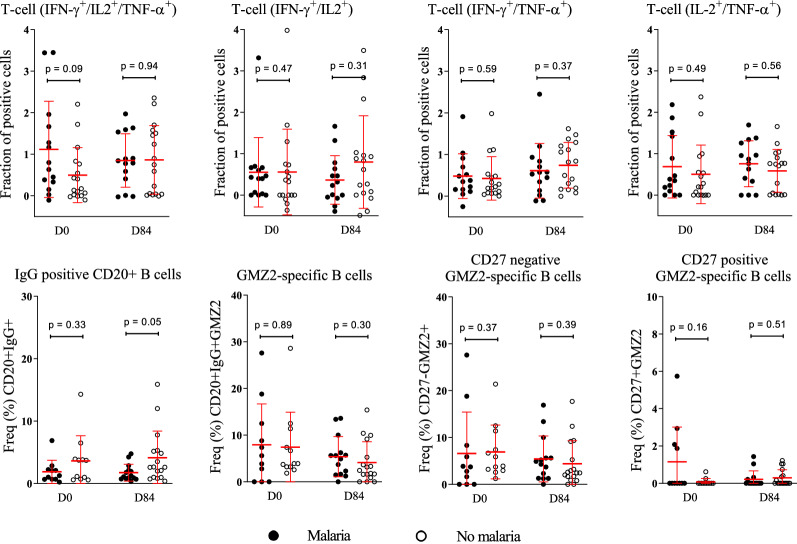
Association between T and B phenotypes with the trial outcome. Dot plot graphs show the relation between pre- and post-immunization GMZ2-specific immune phenotypes regarding presence/absence clinical malaria status after CHMI. Any parasitaemia with symptoms or parasitaemia above 1000 parasites per µl was defined as malaria (black spots), whereas any parasitaemia below the threshold of 1000 parasites / µl with no symptoms (Control) as well as individuals with neither parasitaemia nor symptoms (Protected) are represented by open circles. Comparison of cytokine producing CD4^+^ T-, CD20 + B- and the GMZ2-specific B phenotypes was performed using Mann-Whitney (T cells) or unpaired t-tests (B cells). Data are from a single experiment after several optimization tests, and individual volunteers were measured in separate experiments. Symbols represent individual samples. Red lines represent median values and interquartile range. p value lower than 0.05 is considered significant

**Fig. 5:**
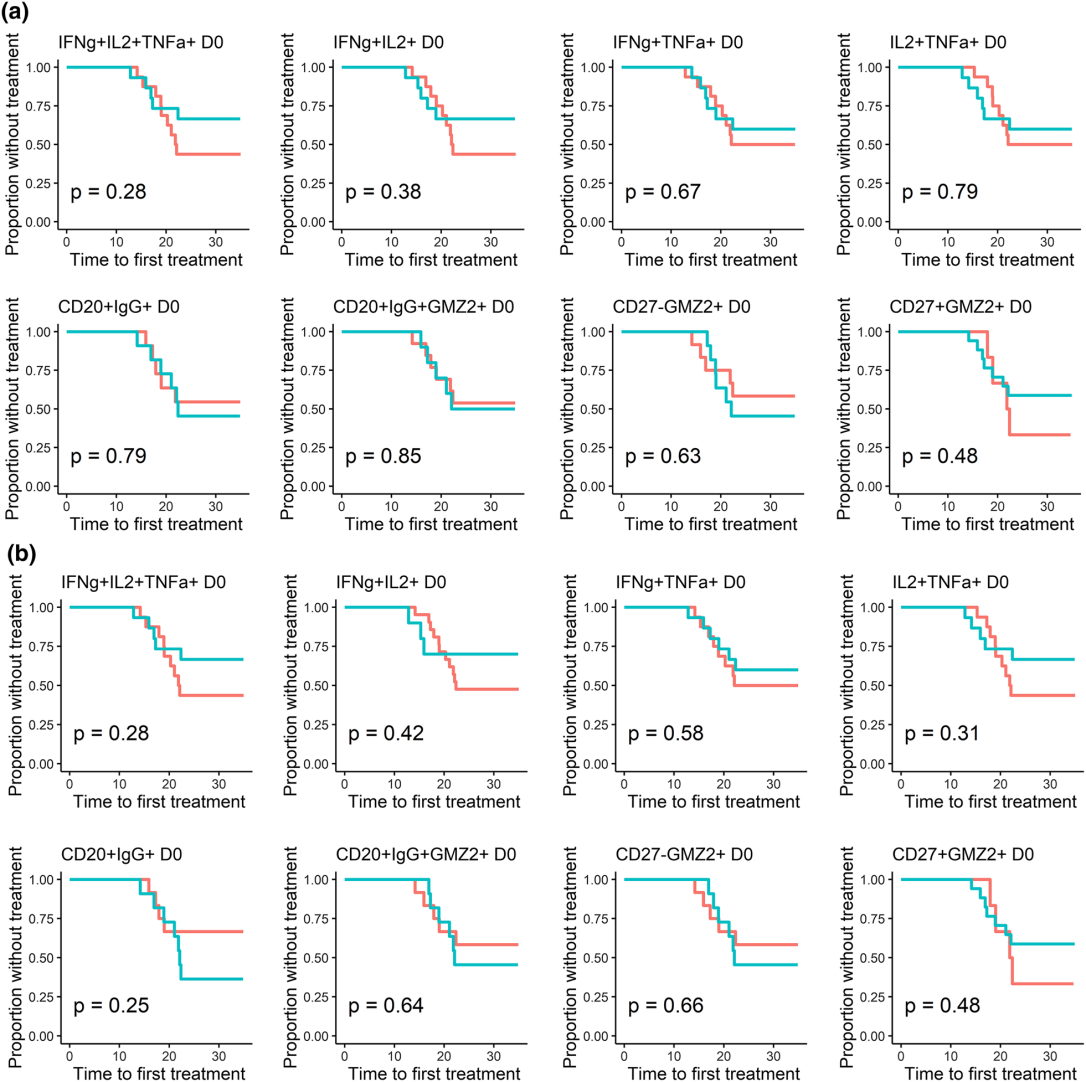
Time to treatment regarding natural acquired immunity before vaccine intervention. Graphs show the time to first malaria treatment regarding the fraction of specific triple and double positive CD4^+^ T, CD20^+^IgG^+^ GMZ2^±^ and GMZ2-specific-CD27^±^ B cells (**a**), or the estimated cell number for each of the same cell phenotypes (**b**) at baseline (D0). Values above the median are represented in red whereas data below the median are shown in blue. The Log-rank test was used to compare the two curves. P value lower than 0.05 is considered significant

Exploratory analyses of data collected at D0 and D84 were done in a subgroup of participants who underwent CHMI to assess whether pre- or post-immunization cellular patterns were associated with protection from experimentally induced malaria [22]. Thereby, higher frequencies of CD20^+^IgG^+^ B cells were found to be associated with protection from parasitaemia after CHMI (Fig. 4). Surprisingly, neither T-stimulated nor GMZ2-specific B cells (Fig. 4). No significant association between the pre-immunization (D0) cell frequencies (Fig. 5a) or the estimated cell counts (Fig. 5b) and the time to malaria treatment after CHMI was observed. In the same way, no significant association between the baseline cytokine producing CD4^+^ T cells and the outcome in CHMI was observed (Table 2).

**Table 2:** Cox proportional analysis pre-immunization

	Risk to be treated after CHMI					
	Cell frequency (Pre-immunization)			Estimated cell number (Pre-immunization)		
	HR	95%CI	p value	HR	95%CI	p value
IFN_γ_^+^IL2^+^TNFα^+^ CD4^+^ T	1.49	0.92–2.40	0.10	1.02	0.99–1.04	0.13
IFN_γ_^+^IL2^+^ CD4^+^ T	0.92	0.52–1.64	0.78	0.98	0.79–1.20	0.86
IFN_γ_^+^TNFα^+^ CD4^+^ T	1.28	0.46–3.55	0.63	0.99	0.95–1.03	0.85
IL2^+^TNFα^+^ CD4^+^ T	1.15	0.58–2.27	0.68	1.00	0.99–1.01	0.56
IgG positive CD20 + B cell	0.85	0.56–1.10	0.16	1.00	0.99–1.00	0.59
GMZ2-specific B cells	1.01	0.94–1.1	0.64	1.00	0.99–1.00	0.07
CD27 negative GMZ2-specific B cells	1.01	0.91–1.12	0.74	1.00	0.99–1.00	0.21
CD27 positive GMZ2-specific B cells	1.39	0.98–1.96	0.06	1.01	0.99–1.02	0.07

Treatment after CHMI was administered to those who developed malaria or to those whose parasitaemia was more than 1000 parasites per µL.

p value was significant when less than 0.05

*CI* confidence interval, *HR* hazard ratio.

At D84, protected volunteers had a higher number of circulating IgG positive CD20^+^ B cells compared to those having malaria although this difference did not reach statistical significance (p = 0.06) (Additional file 1: Fig. S4). Furthermore, an association between higher IgG positive CD20^+^ B frequency and the time to malaria treatment after CHMI (D84) was observed (Additional file 1: Fig. S5a) whereas post-immunization estimated counts (Additional file 1: Fig. S5b) did not. Additionally, Cox regression using post-immunization data showed that the hazard of malaria in CHMI was not significantly associated with the proportion of cells at D84 (Additional file 1: Table S1).

### GMZ2-specific IgG concentration and B cells baseline

**Fig. 6:**
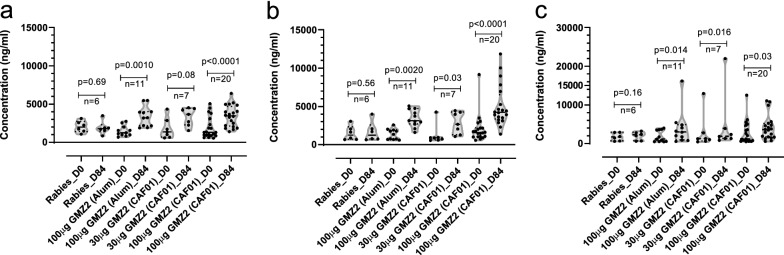
Plasma IgG concentrations against GMZ2. IgG antibody concentration was measured by enzyme-linked immunosorbent assay (ELISA) in all study volunteers against the vaccine immunogen GMZ2 (**a**) as well as the GLURP (**b**) and MSP3 (**c**) fragments. Plasma was collected at days 0 and 84. Every violin plot represents the distribution within each interventional group. Bold lines indicate the median, and dotted lines mean the quartiles. The Wilcoxon matched pairs signed rank test were used to assess vaccine immunogenicity between days 0, and 84. p value lower than 0.05 is considered significant

GMZ-specific antibodies were specifically elicited by GMZ2 vaccination regimen (Fig. 6a–c). The association between B cells and the concentration of anti-GMZ2 IgG was analysed. This was done as anti-GMZ2 IgG concentration at baseline, but not after vaccination was shown to be predictive for CHMI outcome, as was already previously published [22].

**Fig. 7:**
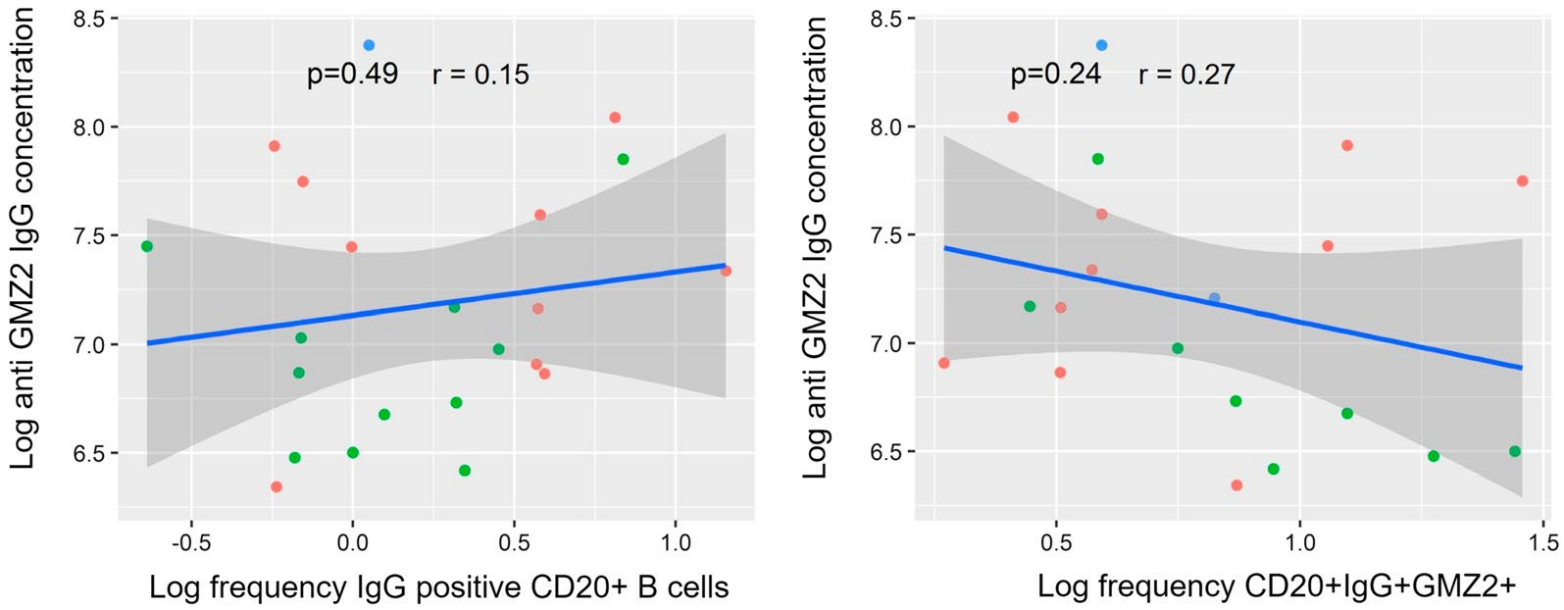
Correlation between GMZ2^−/+^ B cell phenotypes and the anti-GMZ2 IgG concentration at baseline. The association between GMZ no specific CD20^+^IgG^+^ or CD20^+^IgG^+^GMZ2^+^ B cells and the anti-GMZ2 IgG concentration was performed on D0 data using Pearson’s correlation after log transformation. Data were obtained from separate experiments after several optimization tests. Symbols represent individual samples. p value lower than 0.05 is considered significant

GMZ2-specific IgG concentration was not associated with the frequencies of neither the CD20^+^IgG^+^ B or the CD20^+^IgG^+^ GMZ2 + B cells (Fig. 7). Likewise, the estimated B-cell counts circulating in the blood were not associated with the antigen-specific IgG concentration (Additional file 1: Fig. S6).

### Pre-existing antibody response in lifelong malaria-exposed volunteers predicts protection from malaria in CHMI

**Fig. 8:**
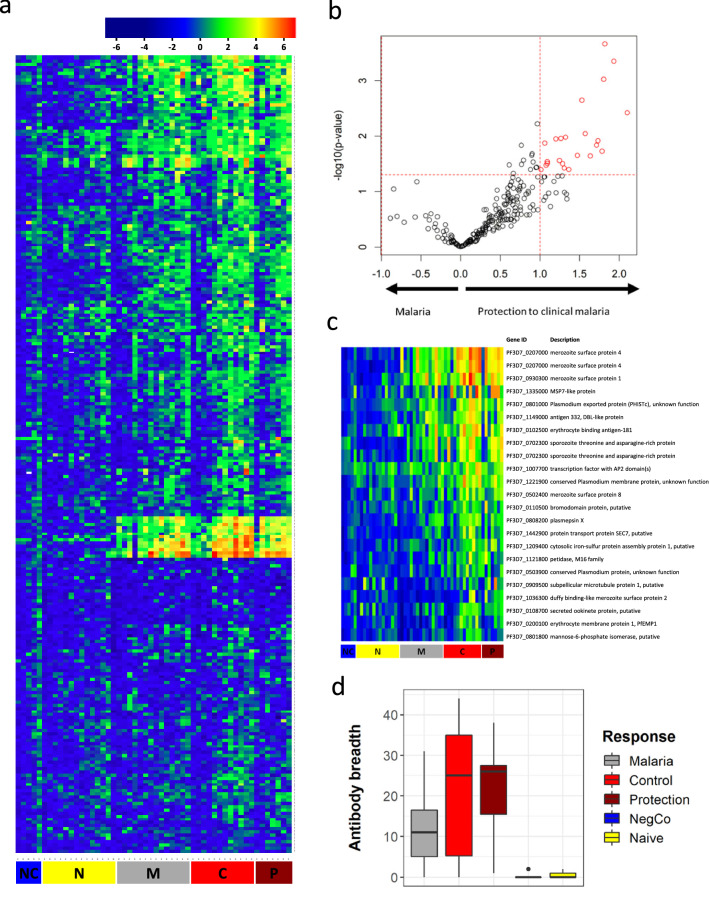
Protein microarray using plasma from volunteers undergoing CHMI. Figure shows the heatmap from protein microarrays in the semi-immune study population as well as a European malaria-naïve control population (**a**). The intensity of antibody responses in the population being protected from clinical malaria and those who developed malaria were compared and shown as volcano plot. The red circles are antigens being at least two-fold higher and significantly upregulated in the respective group (**b**). **c** shows the heatmap in participants having at least two-fold higher antibody response in the protected group vs. the unprotected group, thus showing the raw data of the red dots in Fig. 8b. Gene ID according to PlasmoDB are given. **d** shows the breadth of the antibody response in those who develop malaria (n = 15), those who control the parasitaemia (n = 12), those having full protection (n = 7), in the negative control (1 European malaria-naïve sample, measured in 5 technical replicates) and in European naïve controls (n = 13). Data are from a single experiment after several optimization tests, and individual volunteers were measured in separate experiments. Differential antibody recognition in the different allocated study outcomes was analysed by Student’s t-test. p value less than 0.05 is considered as statistically significant. *NC* Negative control, *N* malaria-naïve subjects, *M* subjects having clinical symptoms of malaria after CHMI, *C* subjects controlling parasitaemia, *P* subjects fully protected after CHMI

To quantify the immune response against a large set of malaria antigens, protein microarrays were performed in the semi-immune study population as well as a European malaria-naïve control population (Fig. 8a). Mean antibody responses to 86 of the 251 expressed *P. falciparum* antigens were at least two-fold higher and significantly different in the African population compared to the European population. Further analysis within the Gabonese study population revealed that antibody responses were significantly higher in the population protected from clinical malaria (no symptoms and either submicroscopic parasitaemia or no parasitaemia during the 35 days of follow up post-CHMI) than in the population that developed symptoms in CHMI (Fig. 8b). Elevated antibody levels were directed against a range of antigens. The pattern included antigens expressed at various stages of the life cycle and localized at different sites (merozoite surface, erythrocyte membrane, intracellular, sporozoite protein) of the parasite (Additional file 2: Table S2). In total, 23 spots, representing 21 different antigens, were significantly and at least twofold higher recognized by specific antibodies in the protected group vs. the unprotected group. These included well characterized, highly immunogenic antigens that are described as markers for long-term exposure. Some have been investigated as vaccine candidates, such as merozoite surface protein 1 (MSP1), MSP4, MSP8, erythrocyte binding antigen 181 (EBA-181), sporozoite threonine and asparagine-rich protein (STARP). Individual results for the antigens associated with protection against clinical malaria are depicted in Fig. 8c.

Neither GLURP nor MSP3 were contained on the microarray and therefore could not be part of this specific analysis, but these antigens have thoroughly been analysed in previous work by ELISA (Fig. 6) [22]. In addition, the correlation of the antibody response against the vaccine antigens with any of the antigens represented on the protein microarray was investigated (Additional file 3: Table S3). Interestingly, the immune response to MSP3 correlated to several other members of the MSP-family (MSP5, MSP10, MSP11). Also, it is noticeable that the response measured to GMZ2, GLURP and MSP3 also correlated with the antibody response against several PfEMP1 proteins.

Immunity to clinical malaria develops after years of repetitive exposure to the parasite, potentially increasing the breadth of the antigens recognized by the immune system. As expected, volunteers who developed clinical symptoms had a significantly lower breadth of highly recognized antigens than those who controlled parasitaemia. Regardless, the antibody response in this population was still much higher than in European naïve controls (Fig. 8d).

## Discussion

In this exploratory study, the cellular and humoral immune responses to the GMZ2 malaria vaccine candidate adjuvanted with either Alhydrogel or CAF01 was assessed. Likewise, it was hypothesized that vaccine specific CD4^+^ T cell and CD20^+^ IgG^+^ B cell responses are induced and that they are associated with protection against CHMI.

The observation that immunization with GMZ2 adjuvanted with either Alhydrogel or CAF01 did not significantly augment the CD4^+^ T cell proportion was unexpected, however, an increase in the estimated number of GMZ2-stimulated CD4^+^ T cells was detectable (Additional file 1: Fig. 2). The mechanism of action of these two adjuvants is well described and responses should be measurable in healthy adult volunteers [32] although previous studies have been only conducted in animal models [33, 34] and this study is the first one to directly compare both adjuvants in humans. On one hand, it is possible that stoichiometry between adjuvant and vaccine antigen plays a role besides the chemical nature of the adjuvant, since both vaccine formulations have been optimized in animal models before; and an increase in GMZ2-specific antibodies could be observed. On the other hand, another explanation relying on the marked CD4^+^ T cell activity at baseline may provide a scenario where the saturated activation of T cells and/or previous activation of dendritic cells (DCs) before vaccination may reflect the influence of the frequent natural exposure to *P. falciparum* [35]. It has also been shown that elevated levels of CD161^+^CD4^+^ T cells and malaria-specific IFN-γ-production predicted protection against CHMI [36].

The elevated levels of GMZ2-induced IL-10 following vaccination in both GMZ2 and Rabies vaccinees suggest, that immunoregulatory mechanisms were induced by the inflammatory stimulus of vaccination to help dampening proinflammatory responses [37]. Concerning the explored immunophenotypes, it was surprising not to see higher frequencies of circulating GMZ2-specific CD20^+^ IgG^+^ B cells following immunization. In contrast, GMZ2-specific B cells were increased after vaccination in malaria-naïve adults using Alhydrogel-adjuvanted GMZ2 at D84 [5]. Moreover, GMZ2 vaccine seemed to not stimulate higher frequencies of neither the CD27^−^ cluster, theoretically involving both putative atypical (CD21^−^CD27^−^) and activated (CD21^+^CD27^−^) memory B cells, nor the CD27^+^ B cells (putative classical memory B cells). Natural exposure to malaria parasites through repeated infections can induce protective antibodies [38], whilst simultaneously expanding the population of atypical memory B cells [39]. In future clinical trials investigating vaccines in malaria-endemic countries, this should be investigated in more detail.

Conversely, it was found that after immunization, higher frequency of CD20^+^ IgG^+^ B cells was related to the absence of clinical malaria after CHMI. However, a link between the level of GMZ2- specific memory B cells and the concentration of anti-GMZ2 antibodies was not observed; something that has been seen previously with GMZ2 [5] and other vaccines [40, 41]. Likewise, it was not possible to assign any antigen specific immune phenotype with the prevention of clinical malaria.

On the protein microarray, a set of anti-plasmodial antibodies detected before CHMI were associated with protection. The targeted antigens included several biomarkers of exposure [42] and well-described malaria vaccine candidates. Associations between antibody breadth and protection, as well as responses to specific malaria antigens and protection, have been described in malaria-naïve volunteers immunized with sporozoites under chemoprophylaxis [36, 43]. There, either patterns or specific antigens were associated with protection. So far, highly specific biomarkers predicting protection at a general level are still not found, these could be patterns of overall exposure or immune response to specific antigens or domains. Interestingly enough, the pre-existing antibody response against the vaccination antigens at baseline correlated with several markers of long-term exposure to malaria parasites displayed on the microarray. Antigens of the array used in this study were down-selected from previous studies [28, 43–47] and may be further simplified for an assay that predicts protection following natural or artificial exposure to parasites. Interestingly, a higher breadth of antibody responses was also associated with protection. Certainly, this fact reinforces the hypothesis that protection is mediated by individual patterns of *P. falciparum* antigen-recognition rather than a single antigen, although some antigens are more dominant than others. Both antigens contained in GMZ2 – MSP3 and GLURP – are among those dominant ones since baseline activity against them predicts protection against CHMI, as it has been already shown [22].

This study presents some limitations, most importantly small number of volunteers per group, which only allows the detection of large effects. In addition, specific T cell stimulation was done using the vaccine antigen and not peptides or multimers. Despite these limitations, the study provides important insights on the difficulties to develop a malaria vaccine for malaria-endemic regions. Further exploration of the immunological aspects of naturally acquired immunity in endemic regions are a necessary step in the design and clinical development of any future malaria vaccine with an impact where it is most needed [36].

Immunization with GMZ2 formulated with CAF01 or Alhydrogel did not successfully induce robust increases in CD4^+^ T or CD20^+^ IgG^+^ B cell responses. Disappointingly, data concerning the expansion of estimated CD20^+^IgG^+^ B cell counts highlighted that GMZ2-specific cells did not contribute to reduce the risk of clinical malaria in CHMI.

## Conclusion

The GMZ2-reactive T and B cell patterns examined here show the dominant role of naturally acquired immunity in controlling malaria clinical episodes in the high endemic area of Gabon. Therefore, the differences observed in clinical trials in endemic settings compared to malaria-naïve volunteers stress even more the point that inclusion of populations form malaria-endemic areas early in the clinical development is important.

## Supplementary Information

**Additional file 1: Figure S1a**. Gating strategies for cytokine producing CD4^+^ T cell identification. **Figure S1b.** Gating strategy for GMZ2-reactive memory B cell identification. **Figure S2.** Estimated number of CD4^+^ T cells producing cytokines on unstimulated, vaccine antigen GMZ2 and Staphylococcal endoterotoxin B (SEB) stimulated cells following immunization. Symbols represent individual samples in unstimulated, GMZ2 stimulated and SEB-stimulated conditions. All time points per volunteer were measured in a single experiment after several optimization tests, and individual volunteers were measured in separate experiments. Red lines represent median values with interquartile range. p value lower than 0.05 is considered significant. **Figure S3.** Estimated number of B cells with or without GMZ2-reactivity following immunization. Symbols represent individual samples. All time points per volunteer were measured in a single experiment after several optimization tests, and individual volunteers were measured in separate experiments. Red lines represent the median values with interquartile range. p value lower than 0.05 is considered significant. **Figure S4.** Association between pre/post-immunization GMZ2-specific immune cells and trial outcome. Dot plot graphs show the relation between the estimated number of pre/post-immunization GMZ2 stimulated cytokine producing CD4^+^ T cells (upper side), the number of and B cells subsets (bottom side) regarding clinical malaria status after CHMI. Monotone increase of parasitemia with symptoms (Malaria) is represented by black spots. Low oscillating parasitemia with no symptoms (Control) plus individuals with neither parasitemia nor symptoms (Protected) are represented by open circles. Comparison of the cell number of GMZ2 stimulated CD4^+^ T cells, of CD20+ B cells and the GMZ2-specific B subsets was performed using Mann-Whitney (for T cells) or unpaired t-tests (for B cells). Data are from a single experiment after several optimization tests, and individual volunteers were measured in separate experiments. Symbols represent individual samples. **Figure S5.** Post-immunization cell frequencies and the time to treatment after CHMI. Graphs show the time to first malaria treatment regarding the fraction of specific triple and double positive CD4^+^ T cells, total B cells and the CD27^+/-^ cluster subsets of GMZ2-specific within CD20^+^IgG^+^ B cells (a), or the number of GMZ2-stimulated triple and double positive CD4^+^ T cells, and the number of total B cells and different GMZ2^+^B cells (b) at D84. Values above the median are represented in red whereas data below the median are shown in blue. The Log-rank test was used to compare the two curves. p value lower than 0.05 is considered significant. **Figure S6.** Correlation between the estimated number of B cell phenotypes and the anti-GMZ2 IgG concentration at baseline. The association between the estimated number of CD20+IgG+ B cells, the estimated number of GMZ2-specific B cells and the anti-GMZ2 IgG concentration, was performed on D0 data using Pearson’s correlation after log transformation. Data are from a single experiment after several optimization tests, and individual volunteers were measured in separate experiments. Symbols represent individual samples. A p-value less than 0.05 is considered as statistically significant. **Figure S7.** B cell phenotypes frequency following immunization regarding vaccine intervention. Frequencies of CD20^+^IgG positive, CD20^+^IgG^+^GMZ2-specific and GMZ2-specific CD27^+/-^ B cells between D0 and D84 are compared for all volunteers regarding vaccine intervention. Vaccinated subjects with Rabies control vaccine are represented with opened squares. GMZ2 vaccinated discriminate vaccinees receiving 100µg GMZ2-Alhydrogel (opened dots), 30µg GMZ2-CAF01 (grey dots), and 100µg GMZ2-CAF01 (black dots). Wilcoxon test following by Bonferroni correction for multiple comparison is performed to test statistical significance. p value below 0.05 is considered statistically significant. Data are from a single experiment after several optimization tests, and individual volunteers were measured in separate experiments. Symbols represent individual samples. Red lines represent the median values with interquartile range. p value lower than 0.05 is considered significant. **Table S1**. Cox proportional analysis post-immunization

**Additional file 2: Table S2.** Complete list of antigens spotted on the protein microarray.

**Additional file 3**:** Table S3.** The antibody response against the vaccine antigen (GMZ2) or any of the main components (MSP3, GLURP), estimated before vaccination, was tested for correlation with any of the antibody responses, as estimated by the protein microarray before CHMI. The log-transformed ELISA data and the protein microarray data were tested usind Student's t-test for correlation. Antigens with correlations resulting in p-values < 0.05 are shown.

## Data Availability

The datasets generated during and/or analysed during the current study are available from the corresponding author on reasonable request.

## Abbreviations

Alhydrogel: Alhydrogel hydroxide
CAF: Cationic adjuvanted formulation
CD: Cluster of differentiation
CHMI: Controlled human malaria infection
HBSS: Hank’s balanced salt solution
IgG: Immunoglobulin G
IL: Interleukin
IFN-γ: Interferon gamma
PBMC: Peripheral blood mononuclear cells
RPMI: Roswell Park memorial institute medium
Th: T helper
TNF: Tumour necrosis factor
VE: Vaccine efficacy
