# Supplementary material for: Cellular and antibody response in GMZ2-vaccinated Gabonese volunteers in a controlled human malaria infection trial
Source: Malar J. 2022 Jun 17;21:191. doi: 10.1186/s12936-022-04169-8 (PMC9204906; doi:10.1186/s12936-022-04169-8)
Supplement: Supplementary file 1 — Additional file 1: Figure S1a. Gating strategies for cytokine producing CD4+ T cell identification. Figure S1b. Gating strategy for GMZ2-reactive memory B cell identification. Figure S2. Estimated number of CD4+ T cells producing cytokines on unstimulated, vaccine antigen GMZ2 and Staphylococcal endoterotoxin B (SEB) stimulated cells following immunization. Symbols represent individual samples in unstimulated, GMZ2 stimulated and SEB-stimulated conditions. All time points per volunteer were measured in a single experiment after several optimization tests, and individual volunteers were measured in separate experiments. Red lines represent median values with interquartile range. p value lower than 0.05 is considered significant. Figure S3. Estimated number of B cells with or without GMZ2-reactivity following immunization. Symbols represent individual samples. All time points per volunteer were measured in a single experiment after several optimization tests, and individual volunteers were measured in separate experiments. Red lines represent the median values with interquartile range. p value lower than 0.05 is considered significant. Figure S4. Association between pre/post-immunization GMZ2-specific immune cells and trial outcome. Dot plot graphs show the relation between the estimated number of pre/post-immunization GMZ2 stimulated cytokine producing CD4+ T cells (upper side), the number of and B cells subsets (bottom side) regarding clinical malaria status after CHMI. Monotone increase of parasitemia with symptoms (Malaria) is represented by black spots. Low oscillating parasitemia with no symptoms (Control) plus individuals with neither parasitemia nor symptoms (Protected) are represented by open circles. Comparison of the cell number of GMZ2 stimulated CD4+ T cells, of CD20+ B cells and the GMZ2-specific B subsets was performed using Mann-Whitney (for T cells) or unpaired t-tests (for B cells). Data are from a single experiment after several optimization tests, [file 12936_2022_4169_MOESM1_ESM.docx]

**Additional file 1: information**


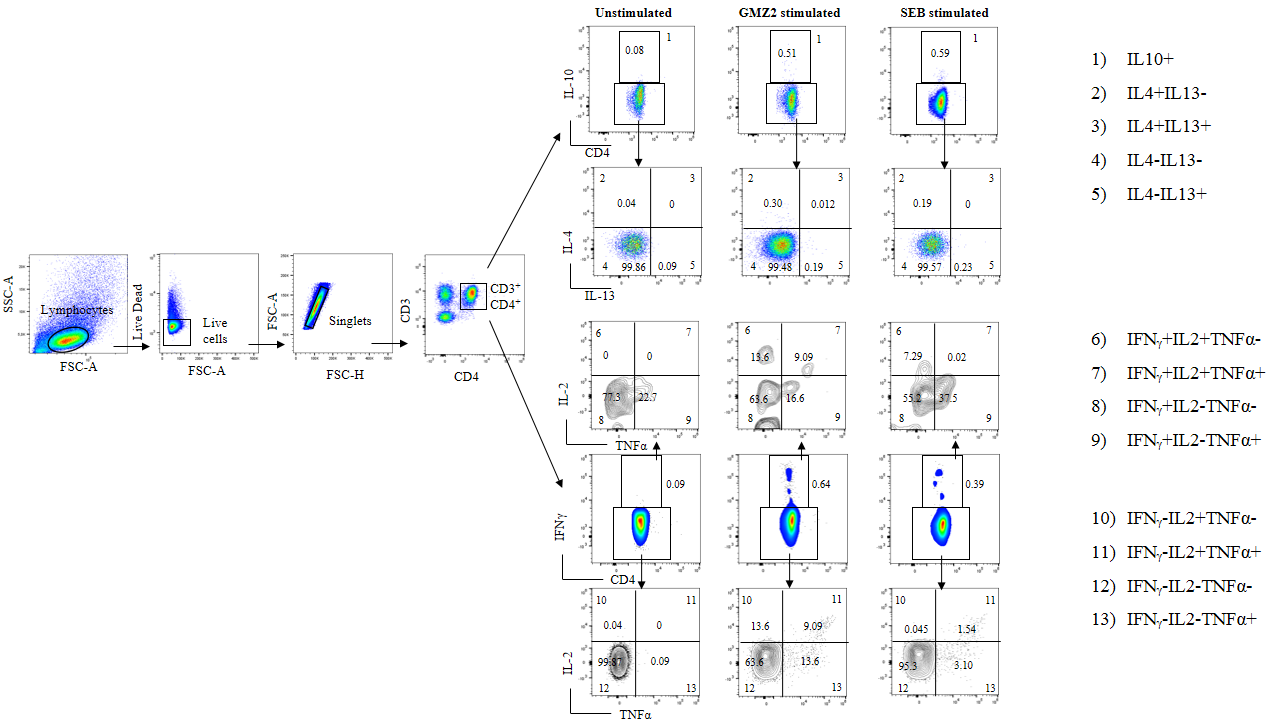


Additional file 1: Figure 1a: Gating strategies for cytokine producing CD4^+^ T cell identification


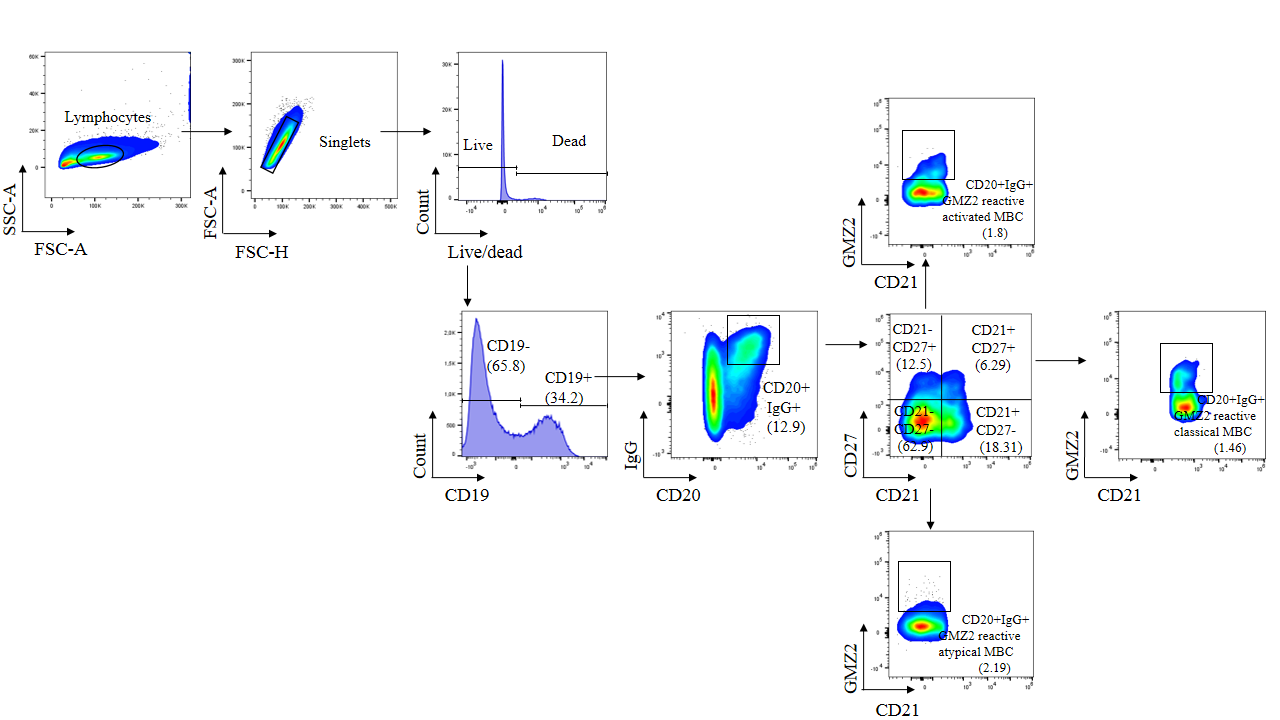


Additional file 1: Figure 1b: Gating strategy for GMZ2-reactive memory B cell identification

**Additional file 1: Figure 1.** **Flow cytometry gating strategy for the cytokine producing CD4+ T cell and B cell populations**

Isolated PBMCs were stimulated with either medium alone, the vaccine antigen GMZ2, or SEB as positive control. Thereafter, intracellular cytokine staining was performed, and the cells measured by flow cytometry. The frequencies of triple, double and single pro-inflammatory positive cytokine producing CD4^+^ T cells were obtained after gating either the CD4^+^IFN-γ^+^ or the CD4^+^IFN-γ^-^ populations on unstimulated, GMZ2 stimulated and SEB stimulated cells. The anti-inflammatory IL-10 producing CD4^+^ T cells were obtained after gating in total CD4^+^ T cells population, and IL-4 and IL-13 producing CD4^+^ T cells were identified after gating in CD4^+^IL-10^-^ populations (Additional file 1: Figure 1a). B-cells were measured using cryopreserved PBMCs without additional stimulation. GMZ2-specific B subsets (CD27^+/-^) were identified after gating respectively on CD20^+^IgG^+^ B cell population (Additional file 1: Figure 1b).


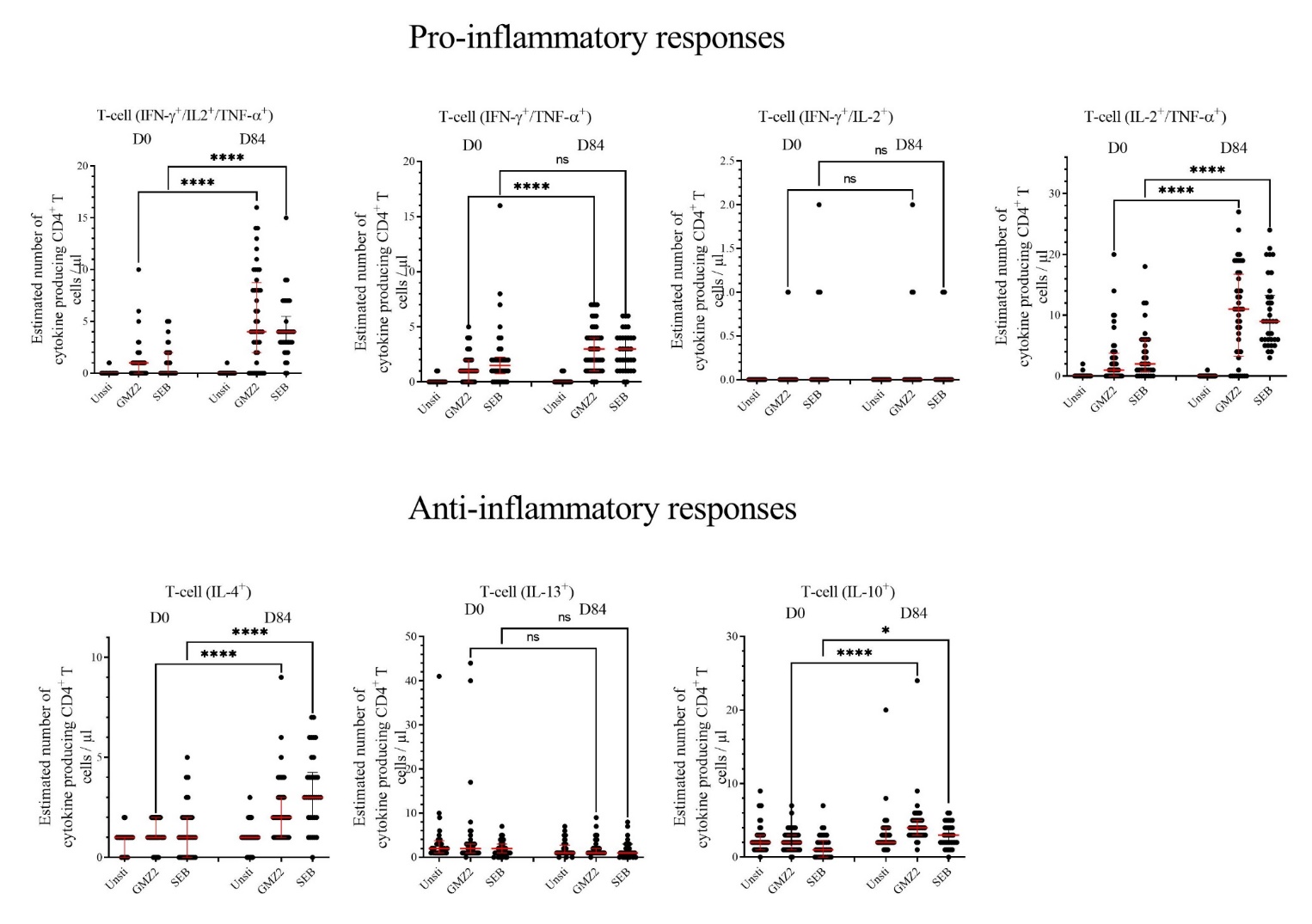


**Additional file 1: Figure 2.** Estimated number of CD4^+^ T cells producing cytokines on unstimulated, vaccine antigen GMZ2 and Staphylococcal endoterotoxin B (SEB) stimulated cells following immunization. Symbols represent individual samples in unstimulated, GMZ2 stimulated and SEB-stimulated conditions. All time points per volunteer were measured in a single experiment after several optimization tests, and individual volunteers were measured in separate experiments. Red lines represent median values with interquartile range. p value lower than 0.05 is considered significant.


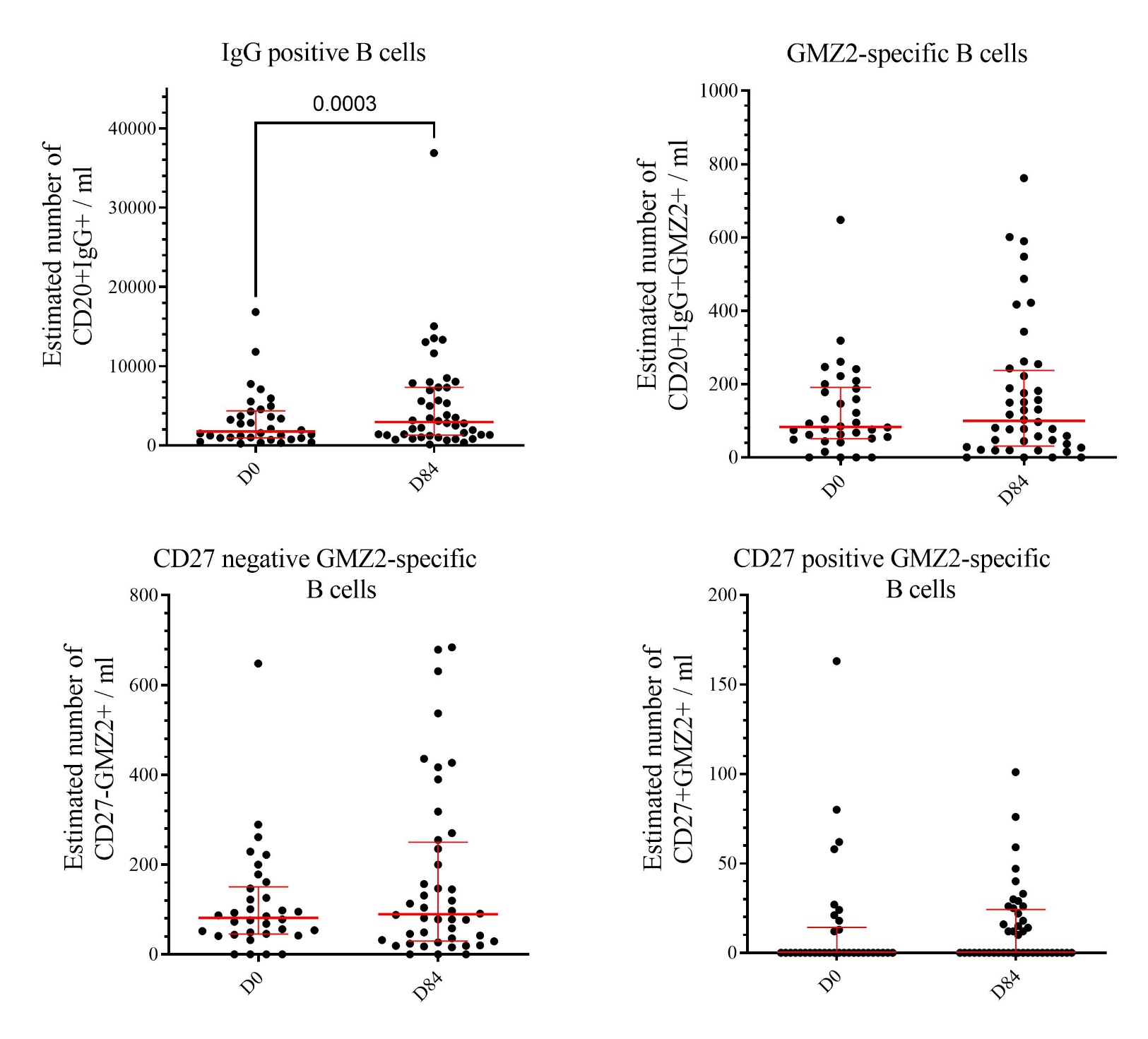


**Additional file 1: Figure 3.** Estimated number of B cells with or without GMZ2-reactivity following immunization. Symbols represent individual samples. All time points per volunteer were measured in a single experiment after several optimization tests, and individual volunteers were measured in separate experiments. Red lines represent the median values with interquartile range. p value lower than 0.05 is considered significant.


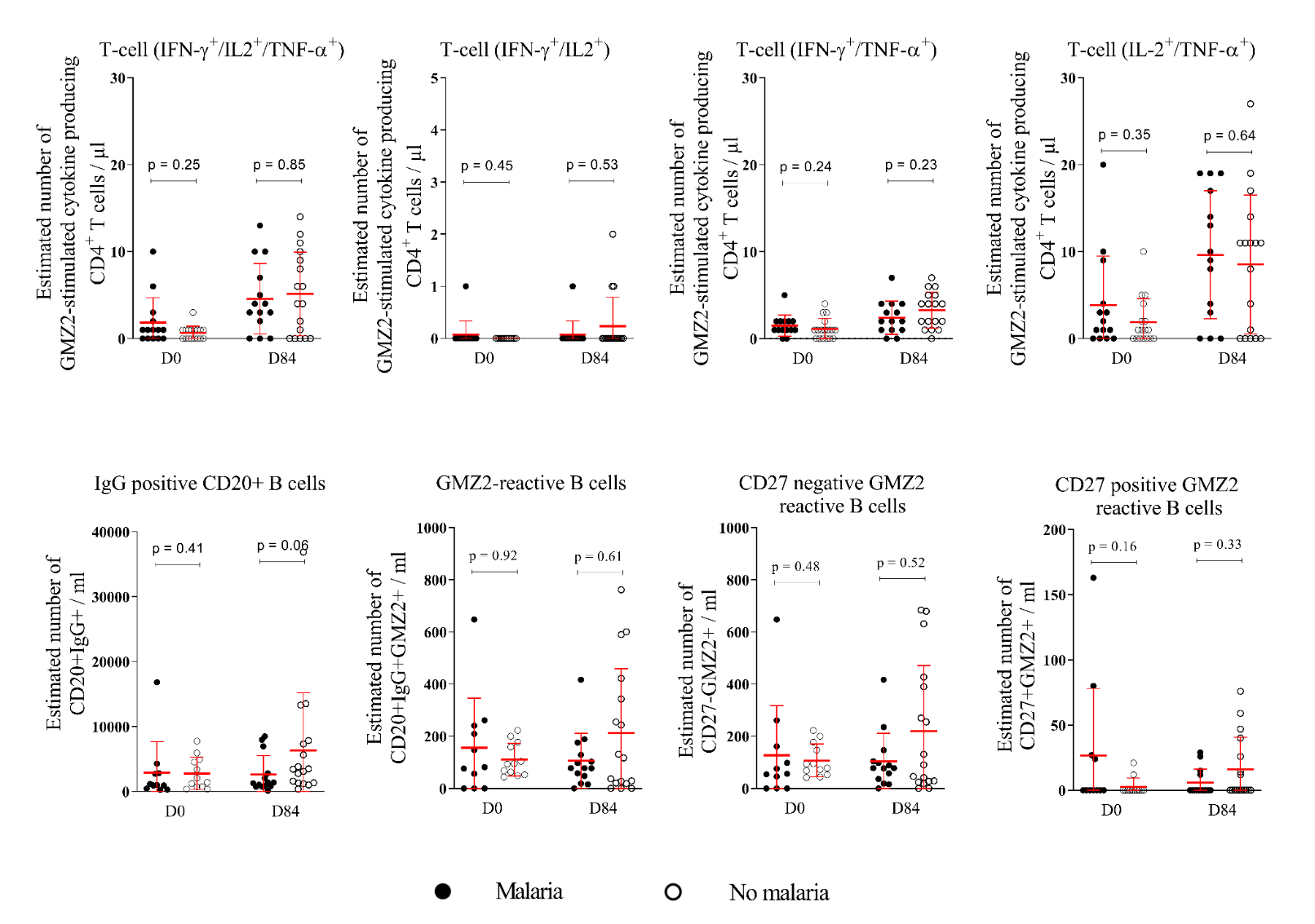


**Additional file 1: Figure 4.** Association between pre/post-immunization GMZ2-specific immune cells and trial outcome.

Dot plot graphs show the relation between the estimated number of pre/post-immunization GMZ2 stimulated cytokine producing CD4^+^ T cells (upper side), the number of and B cells subsets (bottom side) regarding clinical malaria status after CHMI. Monotone increase of parasitemia with symptoms (Malaria) is represented by black spots. Low oscillating parasitemia with no symptoms (Control) plus individuals with neither parasitemia nor symptoms (Protected) are represented by open circles. Comparison of the cell number of GMZ2 stimulated CD4^+^ T cells, of CD20+ B cells and the GMZ2-specific B subsets was performed using Mann-Whitney (for T cells) or unpaired t-tests (for B cells). Data are from a single experiment after several optimization tests, and individual volunteers were measured in separate experiments. Symbols represent individual samples.

Red lines represent the median values with interquartile range. p value lower than 0.05 is considered significant.


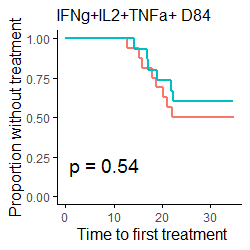

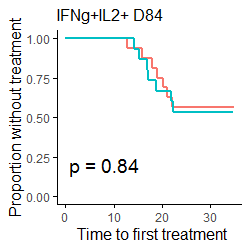

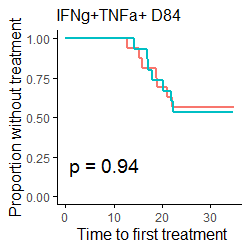

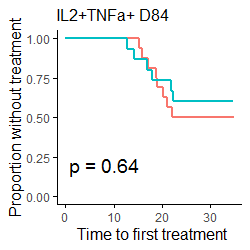


a


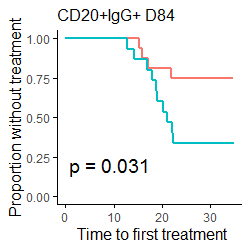

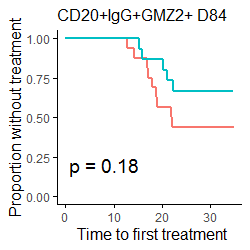

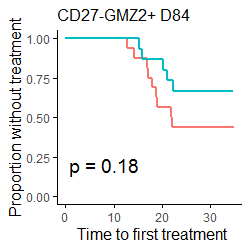

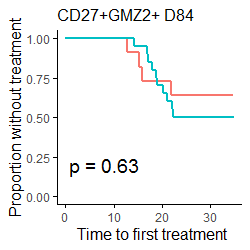


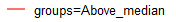

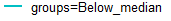


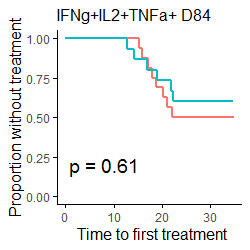

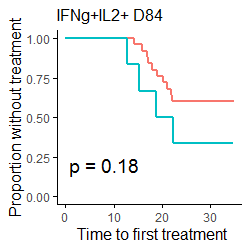

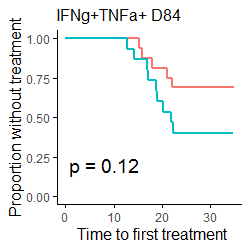

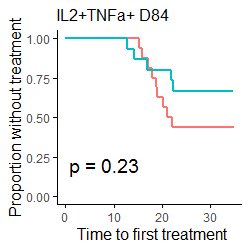


b


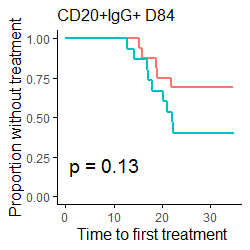

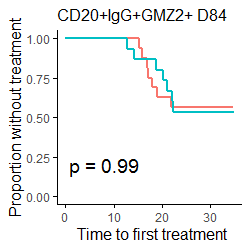

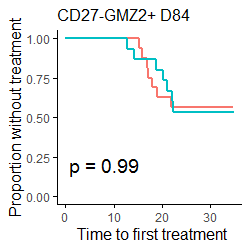

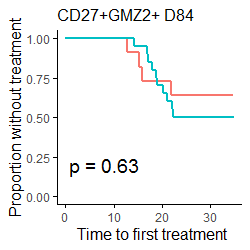


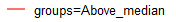

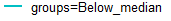


**Additional file 1: Figure 5.** Post-immunization cell frequencies and the time to treatment after CHMI

Graphs show the time to first malaria treatment regarding the fraction of specific triple and double positive CD4^+^ T cells, total B cells and the CD27^+/-^ cluster subsets of GMZ2-specific within CD20^+^IgG^+^ B cells (a), or the number of GMZ2-stimulated triple and double positive CD4^+^ T cells, and the number of total B cells and different GMZ2^+^B cells (b) at D84. Values above the median are represented in red whereas data below the median are shown in blue. The Log-rank test was used to compare the two curves. p value lower than 0.05 is considered significant.


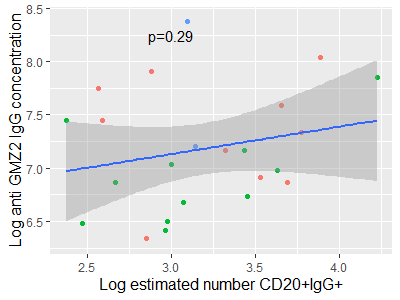

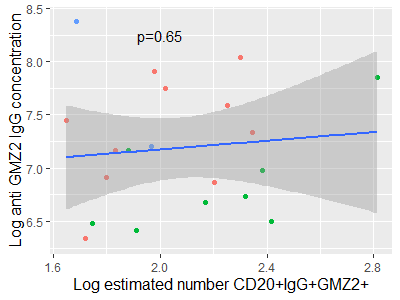


**Additional file 1: Figure 6**. Correlation between the estimated number of B cell phenotypes and the anti-GMZ2 IgG concentration at baseline.

The association between the estimated number of CD20+IgG+ B cells, the estimated number of GMZ2-specific B cells and the anti-GMZ2 IgG concentration, was performed on D0 data using Pearson’s correlation after log transformation. Data are from a single experiment after several optimization tests, and individual volunteers were measured in separate experiments. Symbols represent individual samples. A p-value less than 0.05 is considered as statistically significant.


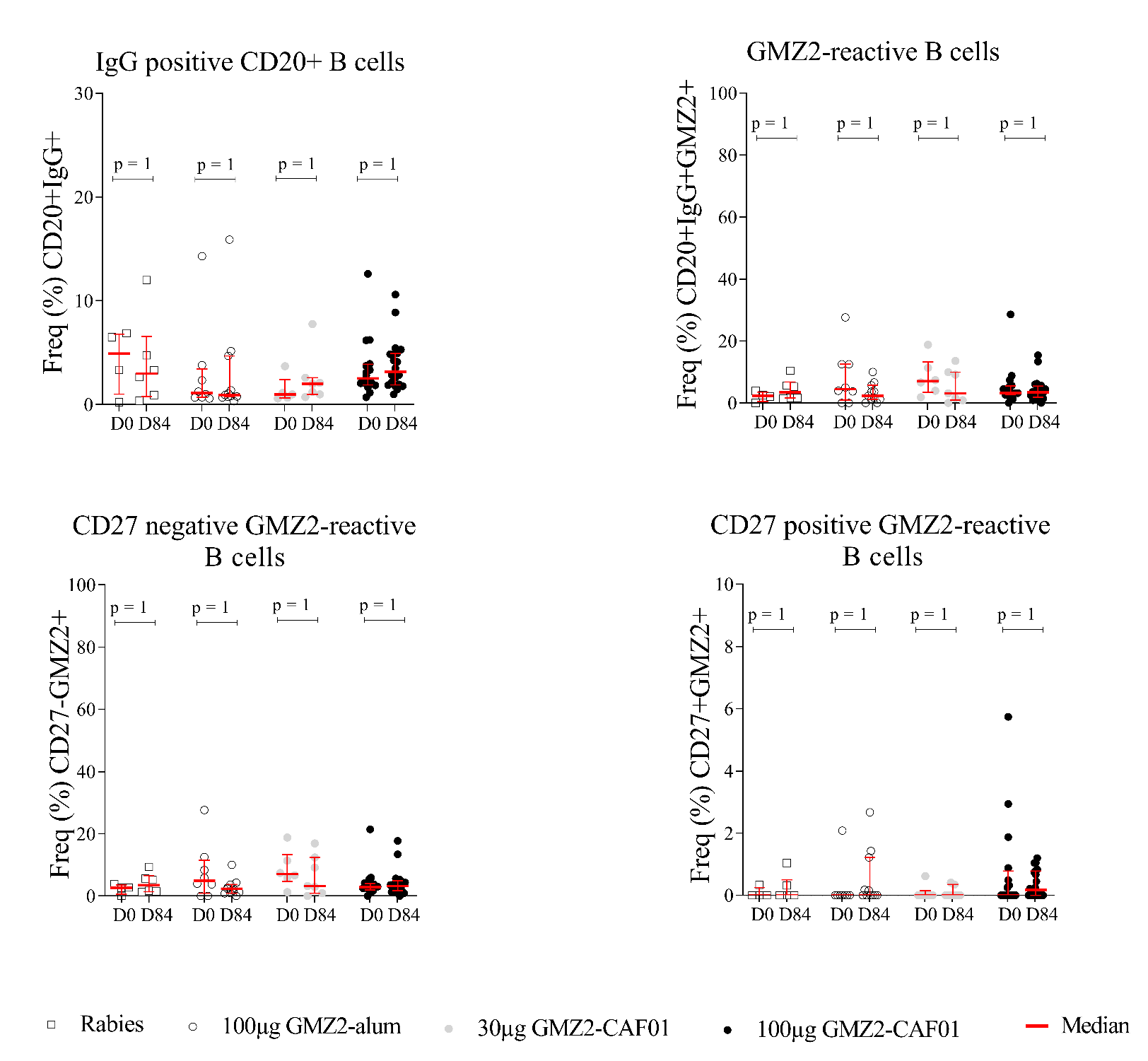


**Additional file 1: Figure 7.** B cell phenotypes frequency following immunization regarding vaccine intervention

Frequencies of CD20^+^IgG positive, CD20^+^IgG^+^GMZ2-specific and GMZ2-specific CD27^+/-^ B cells between D0 and D84 are compared for all volunteers regarding vaccine intervention. Vaccinated subjects with Rabies control vaccine are represented with opened squares. GMZ2 vaccinated discriminate vaccinees receiving 100µg GMZ2-Alhydrogel (opened dots), 30µg GMZ2-CAF01 (grey dots), and 100µg GMZ2-CAF01 (black dots). Wilcoxon test following by Bonferroni correction for multiple comparison is performed to test statistical significance. p value below 0.05 is considered statistically significant. Data are from a single experiment after several optimization tests, and individual volunteers were measured in separate experiments. Symbols represent individual samples. Red lines represent the median values with interquartile range. p value lower than 0.05 is considered significant.

Additional file 1: Table 1. Cox proportional analysis post-immunization.

|  | Risk to be treated after CHMI | | | | | | |
| --- | --- | --- | --- | --- | --- | --- | --- |
|  |  |  |  |  |  |  |  |
|  | Cell frequency (Post-immunization) | | |  | Cell absolute number (Post-immunization) | | |
|  | HR | 95%CI | p value |  | HR | 95%CI | p value |
| IFNᵧ^+^IL2^+^TNFα^+^ CD4^+^ T | 0.70 | 0.29 – 1.68 | 0.42 |  | 1.00 | 0.99 – 1.01 | 0.92 |
| IFNᵧ^+^IL2^+^ CD4^+^ T | 0.64 | 0.31 – 1.31 | 0.23 |  | 0.80 | 0.61 – 1.04 | 0.09 |
| IFNᵧ^+^TNFα^+^ CD4^+^ T | 0.54 | 0.14 – 2.15 | 0.38 |  | 0.98 | 0.96 – 1.01 | 0.28 |
| IL2^+^TNFα^+^ CD4^+^ T | 1.84 | 0.45 – 7.47 | 0.39 |  | 1.00 | 0.99 – 1.00 | 0.30 |
| IgG positive CD20+ B cells | 0.76 | 0.55 – 1.06 | 0.11 |  | 0.99 | 0.99 – 1.00 | 0.21 |
| GMZ2-specific B cells | 1.06 | 0.94 – 1.18 | 0.31 |  | 0.99 | 0.99 – 1.00 | 0.21 |
| CD27 negative GMZ2-specific B cells | 1.04 | 0.94 – 1.15 | 0.42 |  | 0.99 | 0.99 – 1.00 | 0.18 |
| CD27 positive GMZ2-specific B cells | 0.78 | 0.20 – 3.09 | 0.73 |  | 0.97 | 0.94 – 1.01 | 0.23 |

**Additional file 1: Table 1**. Cox proportional analysis assessing the association between post-immunization CD4^+^ T cell, B cell responses and the risk of requiring malaria treatment after controlled human malaria infection. Treatment after CHMI was administered to those who developed malaria or to those whose parasitemia was more than 1000 parasites per µL. CI = confidence interval, HR = hazard ratio. The HR is adjusted by the baseline cell frequency. p value lower than 0.05 is considered significant.
